# Task Allocation Under High Workload: A Qualitative Study of Nurses’ Fairness Experiences in Chinese Tertiary Grade A Hospitals

**DOI:** 10.1155/jonm/2315457

**Published:** 2026-10-18

**Authors:** Yucheng Cao, Lv Wu, Yang Tang, Xu Deng, Tingting Yan, Yu Gao, Shaolong Zhou, Lili Deng

**Affiliations:** ^1^ School of Nursing Guangzhou University of Chinese Medicine Guangzhou 510006 Guangdong China gzucm.edu.cn; ^2^ Qionghai Institute of Nursing Excellence Qionghai 571434 Hainan China; ^3^ School of Nursing Wuhan University Wuhan 430071 Hubei China whu.edu.cn; ^4^ Liaocheng Vocational and Technical College Liaocheng 252000 Shandong China; ^5^ School of Clinical Medicine Yichun University Yichun 336000 Jiangxi China ycu.jx.cn; ^6^ Faculty of Medicine The Chinese University of Hong Kong Shatin, New Territories Hong Kong China cuhk.edu.hk

**Keywords:** nurses’ workload, nursing management, organizational justice, qualitative research, task allocation

## Abstract

**Background:**

Under high‐workload conditions, clinical nurses arrange care with limited time and staffing. Detailed accounts remain limited regarding how nurses evaluate fairness in everyday task allocation and relate these evaluations to their care experiences and views on reform.

**Objective:**

To explore nurses’ fairness experiences in task allocation under high‐workload conditions in Chinese hospitals, including their expectations and concerns about reform.

**Methods:**

This qualitative descriptive study used convenience sampling to recruit 27 female participants from seven tertiary Grade A hospitals in four regions of China. The core sample comprised 24 frontline clinical nurses; three head nurses were supplementary informants. Online semistructured interviews were conducted from April to May 2026. Thematic analysis combined the interview guide’s predefined areas with open coding and comparison of interview data.

**Results:**

Six themes and 16 subthemes were identified. The themes were as follows: (1) fairness judgments in task allocation; (2) fairness experiences and care adequacy; (3) care trade‐offs under resource constraints; (4) emotional and professional experiences; (5) expectations for task allocation reform; and (6) concerns about task allocation reform. Nurses evaluated burden distribution, staff availability, and alignment between effort and rewards. They also described care reduced under time constraints, changes in care priorities, guilt and lingering fear, emotional buildup and relationship strain, and a defensive work orientation. Expectations involved allocation based on actual workload, flexible support during peak periods, and clear allocation and rotation rules; participants feared further increases in work demands and insufficient resources for implementation, and remained cautious about reform outcomes.

**Conclusions:**

Nurses’ fairness experiences concerned task distribution and accompanying support and recognition, alongside concerns about care adequacy. These accounts provide a descriptive basis for considering task allocation changes responsive to nurses’ needs.

**Implications for Nursing Management:**

Nurse managers could consider actual workload, flexible support during peak periods, and clear allocation and rotation rules, with attention to implementation resources and rest.

## 1. Introduction

Clinical nurses have long worked under high‐workload conditions, which have become an important practical issue affecting nursing care quality and hospital operational efficiency [1, 2]. Previous studies have shown that high workload is closely associated with occupational burnout, missed nursing care, and increased patient safety risks [3, 4]. In Chinese tertiary hospitals, patient turnover, high bed occupancy, and the care needs of critically ill patients increase the complexity of nurses’ work [5], while frequent switching between electronic nursing documentation and immediate requests from patients or colleagues adds further demands within shifts [6]. Existing research has mainly explained this issue from the perspectives of staffing shortages and accumulated work stress, with a primary focus on the outcome‐related consequences of high workload [4, 7]. However, workload levels and associated outcomes do not fully capture nurses’ experiences of work allocation, particularly whether they perceive the burdens they undertake as fairly shared. Research on high workload therefore also needs to incorporate an understanding of nurses’ fairness experiences.

An organizational justice perspective provides a conceptual basis for examining the sharing of tasks, risks, and responsibilities, the reasonableness of allocation rules, and the accompanying support and recognition. In qualitative interviews with nurses from six hospitals in Tehran, Iran, Afzali et al. identified unfairness in work division, payments, interpersonal interactions, and evaluation practices as important aspects of nurses’ experiences [8]. Song et al.’s cross‐sectional study of 1136 young nurses from six hospitals in China found a positive association between perceived organizational justice and job performance [9]. Nursing task allocation provides a concrete work context in which nurses experience and evaluate fairness, as it involves both addressing patients’ care needs and sharing workloads among nurses. For example, assigning critically ill patients to experienced nurses on the basis of clinical competence may be reasonable; however, if demanding tasks repeatedly fall to the same nurse without adequate support and recognition, the fairness of such arrangements may be questioned [10]. Understanding fairness experiences therefore requires examining how nurses evaluate the reasons for differentiated task allocation and the support and recognition accompanying these arrangements.

In clinical practice, task allocation directly determines who is responsible for critically ill patients, high‐risk observation, and additional responsibilities [11]. Existing studies commonly use quantitative indicators, such as staffing levels and nurse‐to‐bed ratios, to assess nursing workforce resources and workload. However, these indicators do not fully capture task complexity or the staff actually available for care, and may overlook activities such as communication and coordination that require sustained interaction and time investment [12, 13]. Previous research has connected fairness with rostering and task allocation practices. In a cross‐sectional study of 386 Japanese shift‐working nurses, Kida and Takemura found that unequal work assignments and procedural justice were both associated with turnover intention [14]. In a qualitative study of 17 nurses in Saudi Arabia, Dakhilallah et al. found that participants understood allocation fairness in terms of equitable workload sharing within specific contexts and interpersonal respect, rather than simple numerical equality [15]. Understanding allocation fairness therefore also requires listening to nurses’ detailed accounts of their own workloads and allocation arrangements. In Chinese tertiary Grade A hospitals, high patient volumes, complex care needs, and multiple concurrent demands mean that nurses frequently undertake varied tasks and responsibilities amid busy working conditions. Against this background, gaining a fuller understanding of how Chinese nurses experience and evaluate fairness in task allocation, particularly how they understand the reasonableness of workload sharing, the responsibilities they undertake, and the accompanying support, can help clarify the meaning of organizational justice in everyday nursing work.

Accordingly, this study adopted a qualitative descriptive design and used task allocation as the entry point to explore nurses’ fairness experiences and their views on improving task allocation under high‐workload conditions in Chinese hospitals. Three research questions guided the study: (1) How do nurses understand fairness in nursing work and evaluate the reasonableness of everyday task allocation? (2) What fairness‐related work experiences do nurses report under high‐workload conditions, and how do they perceive the implications of these experiences for themselves, patients, and their teams? (3) What expectations and concerns do nurses have about improving task allocation? This study aimed to provide a basis for improving task allocation and organizational support through nurses’ accounts of work arrangements and fairness experiences.

## 2. Methods

### 2.1. Study Design

This study adopted a qualitative descriptive design and used semistructured individual interviews to collect data, aiming to describe nurses’ fairness experiences under high‐workload conditions, their perceptions related to task allocation, and their expectations and concerns regarding improvements in task allocation. A qualitative descriptive design is suitable for exploring experiential issues in clinical practice that have received limited systematic examination. It supports an approach that remains close to participants’ original accounts and actual work contexts while allowing their experiences and views to be summarized and synthesized. This study was reported in accordance with the Consolidated Criteria for Reporting Qualitative Research (COREQ) [16].

### 2.2. Participants and Sampling Methods

From April to May 2026, convenience sampling was used to recruit nurses for interviews from seven tertiary Grade A hospitals in Chongqing, Zhejiang, Jiangsu, and Guangdong, China. Tertiary Grade A hospitals have large patient volumes and high case complexity, with greater concentrations of critically ill patients and emergency care demands. These characteristics make them suitable settings for examining the complexity and variation of task allocation practices and fairness experiences under high‐workload conditions. These considerations informed the selection of tertiary Grade A hospitals as the study setting.

The inclusion criteria were as follows: (1) holding a registered nurse qualification; (2) currently working in a hospital clinical department and either directly participating in patient care and daily nursing tasks or serving as a head nurse with ward‐level nursing management responsibilities; (3) having worked continuously in the current department for at least 6 months; and (4) being able to describe personal work experiences clearly. Frontline clinical nurses directly involved in patient care constituted the core sample, while head nurses served as supplementary informants in their capacity as nurse managers. The exclusion criteria were as follows: (1) neither directly participating in patient care nor undertaking ward‐level nursing management responsibilities, such as personnel working exclusively in nursing education, administrative affairs, research, or quality control; (2) being a nursing intern or undertaking further clinical training or a short‐term rotation; and (3) not having recently participated in clinical nursing or ward‐level nursing management because of extended leave, sick leave, external training, or other reasons.

Before recruitment, the research team contacted the nursing departments at each study site. After institutional permission was obtained, recruitment information was distributed through the clinical departments. The recruitment notice stated that participation was voluntary and that participation or nonparticipation would not affect work arrangements, performance evaluations, promotion opportunities, or relationships with the department or hospital. Researchers contacted interested individuals directly to explain the study purpose, interview content, scheduling, recording requirements, confidentiality measures, and right to withdraw, seeking to minimize the influence of managerial or departmental relationships on willingness to participate and the views expressed.

Recruitment, data collection, and preliminary analysis proceeded concurrently. After every three interviews, the research team organized the transcripts and conducted initial coding to assess whether the additional data provided new information relevant to the research questions. Data saturation was considered to have been reached when three consecutive interviews generated no new core codes or important themes relevant to the research questions. To confirm this judgment, the research team compared the codes and preliminary thematic interpretations generated from these interviews with the existing coding framework and discussed whether the additional data introduced new meanings or categories or required changes to the developing thematic structure. Recruitment was discontinued once the team confirmed that additional interviews elaborated on existing findings without generating new core codes or important themes. During recruitment, two potential participants declined to participate, and one withdrew before the formal interview. A total of 27 participants completed the interviews, of whom 24 frontline clinical nurses constituted the core sample. Given their ward‐level nursing management responsibilities and knowledge of task allocation and work arrangements, three head nurses were included as supplementary informants to provide additional contextual information. All participants were women and worked in tertiary Grade A hospitals.

### 2.3. Data Collection

Based on a review of relevant literature and clinical practice, the research team drafted an initial semistructured interview guide, which was finalized after multiple rounds of discussion (see Supporting S1 for the semistructured interview guide). The guide mainly covered participants’ basic work situations; their understanding of fairness in nursing work; patient and task allocation within specific shifts and their perceptions of the reasonableness of these arrangements; manifestations of task concentration, responsibility distribution, and reductions in care content under high‐workload conditions; the perceived effects of fair or unfair experiences on nurses, patients, and teams; and expectations and concerns regarding improvements in task allocation. These topics established the main areas of inquiry for data collection and informed the scope of the subsequent analysis.

Before the formal interviews, the research team conducted pilot interviews with two clinical nurses who met the inclusion criteria but were not included in the formal sample, to assess the comprehensibility of question wording, question sequence, interview pace, and interview duration. Based on the feedback, minor adjustments were made to selected expressions and probing strategies.

The interviews were conducted by two researchers who had received training in qualitative research methods. Before the study began, both interviewers underwent standardized training covering the study purpose, use of the interview guide, probing and clarification techniques, online interview communication methods, confidentiality requirements, and standards for recording interview notes. Neither interviewer had a direct supervisory role, responsibility for evaluating participants’ performance, or any other conflict of interest involving the participants. These arrangements helped minimize the influence of role relationships on data collection.

Considering that participants were distributed across different regions and hospitals, one‐to‐one, semistructured in‐depth interviews were conducted online via Tencent Meeting. Interview times were agreed upon by the researchers and participants and were arranged, as far as possible, during participants’ rest periods or nonworking hours. Before each formal interview, the researcher reconfirmed that the participant was in a relatively quiet, private, and undisturbed environment and reminded them to use headphones or choose a relatively private space to reduce external interference and the risk of being identified. Each participant completed one formal interview. Where specific issues required further clarification, supporting confirmation was conducted through WeChat. Each interview lasted approximately 30–60 min.

Before each interview, the researcher explained the study purpose, interview content, recording arrangements, confidentiality principles, and the participant’s right to refuse to answer any question or withdraw from the study at any time, and obtained electronic informed consent. With the participant’s permission, the entire interview was audio‐recorded, while pauses, changes in tone, and key interview points were documented simultaneously. During the interview, the researcher used probing, clarification, summary confirmation, and follow‐up questions based on the participant’s responses to enhance the depth and accuracy of the data while avoiding leading expressions as far as possible.

Within 24 h after each interview, a member of the research team transcribed the audio recording verbatim. During transcription, pauses, repetitions, modal particles, and other expressive features relevant to semantic understanding were preserved as far as possible. Names, hospital names, department names, and other identifiable information were deidentified and replaced with codes. After transcription, the researchers checked each transcript against the original audio recording and sent it to the participant for content verification and necessary revision, to improve the accuracy and completeness of the data.

### 2.4. Data Analysis

This study used thematic analysis to organize and synthesize the interview data, with NVivo 12 used for data management and coding. The interview guide established the main areas of inquiry, within which researchers conducted open coding to identify specific experiences and meanings in participants’ accounts. Theme development combined the guide’s predefined areas with comparison and interpretation of the interview data. Data analysis proceeded concurrently with data collection, and preliminary findings informed further probing and clarification in subsequent interviews.

The specific analytic process was as follows: (1) familiarization with the data: the researchers repeatedly read the complete interview materials and original transcripts to gain an overall understanding of the main narrative threads concerning task allocation, fairness experiences, and related impacts; (2) generation of initial codes: statements or paragraphs expressing a particular experience, view, judgment, or situation were treated as meaning units, and open coding was conducted segment by segment on texts relevant to the research questions; (3) categorization and integration: codes with similar meanings, logical connections, or a shared focus on a particular phenomenon were compared, grouped, and integrated to form preliminary categories; (4) development of candidate themes: Candidate themes were developed from these categories through examination of their relationships, boundaries, and hierarchical structure; (5) review and revision of themes: the research team moved repeatedly between the original data, initial codes, categories, and thematic framework to refine theme names, content, and logical relationships; and (6) finalization of themes: the two researchers compared and discussed their coding and thematic interpretations. When disagreements regarding coding or thematic interpretation could not be resolved through discussion, a third researcher was consulted. Final decisions were reached through discussion and consensus, after which the final thematic framework was established, and representative original quotations were selected to support the presentation of the findings.

Negative case analysis focused on accounts that differed from the preliminary interpretation of a theme. Researchers reviewed the relevant passages and their surrounding context to assess whether the differences reflected variations in work circumstances, different understandings of the issue, or a need to revise the initial interpretation. These accounts were then compared with the evidence supporting the theme to determine whether its scope required qualification, its content required revision, or differing perspectives should be retained within it. The rationale and resulting decisions were documented. For example, the preliminary interpretation was that high workload mainly reduced health education and psychological support, while safety‐related care remained protected because it was prioritized; the differing account was “Before giving the medication, I didn’t really have time to fully check its effects… that day the workload was too heavy and time was tight… afterward I felt a bit guilty, because I knew the care wasn’t really sufficient.” (N2); the contextual explanation and basis for interpretation were that N2 described checks that had not been fully completed under heavy workload and time pressure. This account suggested that prioritizing safety‐related activities did not mean that these activities could always be completed adequately. Reduced care therefore could not be understood as affecting only health education and psychological support; the resulting decision was to retain the subtheme “Care reduced under time constraints” and clarify that affected activities could also include safety‐related checks. The interpretation of “Prioritizing safety and key treatments” was also qualified to distinguish nurses’ ordering of care priorities from the adequate completion of those activities.

### 2.5. Trustworthiness and Rigor

The study drew on Lincoln and Guba’s criteria of credibility, dependability, confirmability, and transferability to support rigor [17]. The two interviewers were master’s students in nursing who had received training in qualitative research methods. Their nursing education provided them with prior knowledge of clinical workload, task allocation, and nursing practice, which facilitated understanding of participants’ accounts but could also influence which aspects of the data they attended to and how those accounts were interpreted. To manage these potential influences, both interviewers used the same semistructured interview guide and based probing, clarification, and summary confirmation on participants’ own accounts while seeking to avoid leading expressions. During analysis, interpretations were repeatedly checked against the original transcripts, and the two researchers compared and discussed their coding and thematic interpretations. Accounts that differed from preliminary interpretations were specifically examined. When the two researchers were unable to reach agreement on a coding or thematic interpretation, a third researcher with a nursing background at the rank of associate research fellow was consulted, and the interpretation was finalized through discussion and consensus. Participants reviewed their transcripts to verify the accuracy and completeness of the interview records. Transcripts, coding records, and records of thematic decisions were retained to make the analytic process traceable. Reporting the study setting, participant characteristics, and analytic procedures provided contextual information for readers to assess the transferability of the findings.

### 2.6. Ethical Considerations

This interview study was approved by the Medical Ethics Committee of Yichun University (approval number: 2026074; effective date: February 1, 2026). Before each interview, the researcher explained the study purpose and content, interview format, potential risks, expected benefits, recording arrangements, scope of data use, and participants’ rights, and obtained electronic informed consent. Participants were free to decline to answer any question or withdraw from the study at any time before or during the interview without providing a reason.

Given that the study involved participants’ subjective experiences of task allocation, fairness perceptions, and organizational functioning, participants might have concerns about expressing their views and face a potential risk of being identified. Throughout data collection, transcription, storage, and presentation of the findings, the research team therefore sought to avoid retaining unnecessary identifiable information and deidentified or obscured details relating to hospitals, departments, and individuals. All audio recordings, transcripts, and analytic materials were stored on password‐protected, encrypted computers accessible only to members of the research team. These materials will be retained for 3 years in accordance with ethical requirements and then destroyed as required.

## 3. Results

### 3.1. General Characteristics of the Interviewed Nurses

A total of 27 nurses were included in this study. All were female and worked in tertiary Grade A hospitals. The largest proportion of participants had 5 years or less of work experience (37.0%), followed by those with more than 15 years of experience (29.6%). In terms of professional title, nurses‐in‐charge accounted for the largest proportion (48.1%). In terms of position, most participants were frontline clinical nurses (88.9%). Regarding educational background, most held a bachelor’s degree (59.3%), followed by a master’s degree (37.0%). Participants were distributed across departments including nephrology/hemodialysis centers, urology, intensive care units, emergency departments, surgical departments, cardiology, and geriatric/chronic disease outpatient clinics (see Table 1).

**TABLE 1:** General characteristics of the interviewed nurses (*n* = 27).

Variable	Category	*N*	%
Gender	Female	27	100.0
	Male	0	0.0

Years of work experience	≤ 5	10	37.0
	6–10	3	11.1
	11–15	6	22.2
	> 15	8	29.6

Professional title	Nurse	2	7.4
	Senior nurse	6	22.2
	Nurse‐in‐charge	13	48.1
	Associate chief nurse	6	22.2

Position	Frontline clinical nurse	24	88.9
	Head nurse	3	11.1

Educational background	Bachelor’s degree	16	59.3
	Master’s degree	10	37.0
	Doctoral degree	1	3.7

Department	Nephrology/hemodialysis center	4	14.8
	Urology	2	7.4
	Intensive care unit (ICU)	5	18.5
	Emergency department	4	14.8
	Surgery (neurosurgery, orthopedics, thoracic surgery, and general surgery)	6	22.2
	Cardiology	2	7.4
	Geriatric/chronic disease outpatient clinic	4	14.8

Region	Zhejiang	4	14.8
	Guangdong	7	25.9
	Jiangsu	7	25.9
	Chongqing	9	33.3

*Note:* The mean years of work experience was 10.56 years, with a standard deviation of 6.51 years.

### 3.2. Identified Themes

Analysis of the interview data identified six themes concerning task allocation, care delivery, and work experiences under high‐workload conditions (Table 2): (1) Fairness judgments in task allocation; (2) Fairness experiences and care adequacy; (3) Care trade‐offs under resource constraints; (4) Emotional and professional experiences; (5) Expectations for task allocation reform; and (6) Concerns about task allocation reform.

**TABLE 2:** Themes, subthemes, and representative quotations.

Theme	Subtheme	Representative quotations
1. Fairness judgments in task allocation	1.1 Demanding tasks concentrated among experienced nurses	“Core staff not only do clinical work but also take on roles like team leader and quality control… so their workload is obviously heavier than others.” (N15) “To be honest, pushing the capable ones harder is pretty common. Those who are more capable and responsive… are more likely to be given heavier tasks… but it can’t go on indefinitely.” (N18)
	1.2 Limited staff availability	“At night shift handover, afternoon infusions aren’t even finished, new orders keep coming… and staffing is already cut in half… tasks pile up in the gaps between shifts, so it looks like there are people… but actually there aren’t enough.” (N16) “In the infusion room… there are only two people, but since some are newly rotating, they also need supervision… so it looks like four people on paper, but teaching also takes energy… and we’re still constantly busy.” (N11)
	1.3 Workload overlooked by existing indicators	“In nephrology wards, the nurse‐to‐bed ratio looks okay, but bedside CRRT requires three shifts… on top of the regular night shift workload… staffing is effectively doubled in consumption… but this pressure isn’t directly reflected.” (N1, head nurse) “…when the workload increases… it’s not just about having more patients. It’s more patients arriving at once… more procedures… and more coordination and communication.” (N27)

2. Fairness experiences and care adequacy	2.1 Alignment between effort and rewards	“In big hospitals, there are more patients and higher demands, so the pressure is already high, but the income and returns aren’t necessarily proportional. The hospital is also controlling labor costs… from a nurse’s perspective… effort and return don’t match.” (N14) “…after a night shift, you’re already exhausted, and then the head nurse assigns extra work at the last minute… you end up leaving very late… and still have to keep going, and there’s no corresponding compensation.” (N2)
	2.2 Care reduced under time constraints	“Health education and psychological support are the first to get squeezed. When it’s busy, we can only cover the key points… very briefly… so the completeness of care patients receive definitely differs.” (N19) “…the things that pile up most… are patient education and documentation. When patients or families have more questions, education takes more time… so naturally, the care isn’t sufficient.” (N24)

3. Care trade‐offs under resource constraints	3.1 Prioritizing safety and key treatments	“…we deal with the most urgent things first, like stabilizing lines, pain, bleeding risks… other care has to be pushed back.” (N6) “In my mind… there’s always a priority order—patient safety comes first… documentation and reports come last.” (N14)
	3.2 Unexpected tasks and task reordering	“When supervisors suddenly ask for materials, summaries, inspections, or complaint responses… the whole workflow gets disrupted… we have to reorder tasks and work overtime to catch up.” (N18) “You may have a lot planned, but patients can change anytime… you keep getting interrupted, going back to what you were doing, and then getting interrupted again.” (N20)

4. Emotional and professional experiences	4.1 Guilt and lingering fear	“After being interrupted… you suddenly think… did I miss something?… you feel relieved but also scared… wondering if something was overlooked.” (N7) “…I was quite scared afterward… if it had been discovered any later, the risk would’ve been huge.” (N3)
	4.2 Emotional buildup and relationship strain	“…when it’s been this exhausting and rushed for a long time… coordination gets worse, and over time the team atmosphere is affected.” (N17) “After arguments during communication, I end up holding it in… and the emotions can affect patients, colleagues… even family and friends.” (N15)
	4.3 Defensive work orientation	“…you just try to avoid problems, it’s hard to aim for better care experiences… it becomes a form of self‐protection.” (N9) “…being in a constant state of tension and anxiety… you get more irritable and self‐critical, and eventually you just focus on doing what you’re supposed to do.” (N17)

5. Expectations for task allocation reform	5.1 Allocation based on actual workload	“…you have to look not only at staffing numbers but also at patients’ conditions and the actual work… you can’t rely on surface numbers alone.” (N23) “…patient numbers, illness severity, proportion of high‐risk patients… should carry more weight… holidays, consecutive working days, and special situations should also be considered.” (N1, head nurse)
	5.2 Flexible support during peak periods	“Another infusion room nurse had to respond to two emergency calls… staffing was suddenly pulled away—during peak times, whether there’s support really matters…” (N11) “…ideally, you finish your tasks within the shift, but still keep 10%–20% flexibility to deal with unexpected situations.” (N5, head nurse)
	5.3 Clear allocation and rotation rules	“Rules should be transparent, and rotations should follow a cycle—this really improves the sense of fairness.” (N13) “Fairness means clear rotation—don’t always put the same people under pressure. Stable and predictable arrangements are enough.” (N10)
6. Concerns about task allocation reform	6.1 Further increases in work demands	“I worry that reform will turn into new assessments and requirements… the workload doesn’t get lighter, but actually increases… more pressure.” (N20) “I’m afraid reform will become a performance tool—just looking at numbers, not reality.” (N13)
	6.2 Insufficient resources for implementation	“The biggest issue… is ongoing staffing cuts… clinical pressure and workload keep increasing. If this doesn’t change, reforms are hard to implement.” (N19) “In reality… labor costs are being controlled, resources are insufficient… so even if reform says to add staff, it’s hard to actually do it.” (N12, head nurse)
	6.3 Caution about reform outcomes	“We try to get through things without adding extra shifts… making sure people who are off can actually rest… so with reform, we first want to see if it will push people harder.” (N5, head nurse) “My first reaction is concern… the biggest worry is more work after reform… but at the same time, I hope risks become more controllable.” (N26)

*Note:* N1, N5, and N12 are head nurses; the other participants are frontline clinical nurses.

### 3.3. Theme 1: Fairness Judgments in Task Allocation

Participants linked their fairness judgments to three aspects of everyday task allocation: how demanding tasks were distributed, whether scheduled staff were actually available to provide care, and whether existing indicators reflected the work undertaken. These accounts situated fairness concerns within specific arrangements for distributing burdens, providing support, and recognizing work.

#### 3.3.1. Subtheme 1.1: Demanding Tasks Concentrated Among Experienced Nurses

Participants described a tendency for experienced nurses to be assigned patients with complex conditions, unexpected tasks, and additional responsibilities. They linked these arrangements to experienced nurses’ ability to manage clinical risks, while also questioning the repeated concentration of demanding tasks on the same individuals. Their concerns focused on the continued accumulation of burdens and limited opportunities for rotation.

*“More experienced nurses… are assigned to more complex and heavier groups because they handle emergencies and risks more steadily… so the heavy work keeps falling on the same people.” (N16)*

#### 3.3.2. Subtheme 1.2: Limited Staff Availability

Nurses described a gap between the number of staff on the roster and the staff time actually available for direct patient care. Teaching responsibilities, non‐nursing tasks, and unexpected demands consumed part of this capacity, while staffing was particularly strained during night shifts and handovers. Participants experienced this gap as insufficient support for completing their assigned work.

*“…when we can’t keep up, it’s mostly because a lot of non-nursing tasks take up time… and with sudden situations, it may look like we have enough staff, but in reality, it’s not enough at all.” (N21)*

#### 3.3.3. Subtheme 1.3: Workload Overlooked by Existing Indicators

Nurses described communication, coordination, explanations, and other time‐consuming work as insufficiently reflected in existing indicators. They linked the limited recognition of this work to a sense that their actual burdens were overlooked. A head nurse added a perspective on workload assessment, noting that the additional staffing demands of bedside continuous renal replacement therapy (CRRT) were not adequately reflected in the nurse‐to‐bed ratio. Together, these accounts concerned whether actual work burdens were adequately captured in workload assessment and recognition.

*“A lot of work like communication, coordination, running errands, and explaining things isn’t reflected in traditional indicators… and this kind of invisible burden also makes it feel unfair.” (N4)*

### 3.4. Theme 2: Fairness Experiences and Care Adequacy

Participants expressed judgments about the fairness of their own work arrangements alongside concerns about the adequacy of patient care. Fairness for nurses concerned whether the tasks, risks, and additional responsibilities they undertook were matched by rotation opportunities, support, compensation, and recognition. Care adequacy concerned whether patients’ needs for information, communication, and psychological support were sufficiently met. Both concerns arose in accounts of busy shifts and reflected nurses’ perceptions of their work arrangements and care delivery.

#### 3.4.1. Subtheme 2.1: Alignment Between Effort and Rewards

Nurses’ fairness judgments focused on whether the burdens they undertook were matched by corresponding rewards and support. They considered demanding tasks, high‐risk tasks, and additional responsibilities alongside rotation opportunities, compensation, and recognition. Participants linked a persistent mismatch between work demands and organizational responses to their feelings of unfairness.

*“Heavier, high-risk tasks do need someone to take on, but there should be rotation and proper compensation… otherwise… it’s not fair.” (N24)*

#### 3.4.2. Subtheme 2.2: Care Reduced Under Time Constraints

Nurses described having limited time for psychological support, health education, and detailed explanations during busy shifts. They sometimes had to shorten conversations and cover only essential information, leaving some needs insufficiently addressed. These accounts concerned care that was reduced, delayed, or left incomplete and nurses’ perceptions that patients’ care needs had not been fully met.

*“When it gets too busy, psychological support is the first to be squeezed… there’s no time to sit down and talk properly… so I do feel the care patients receive is not sufficient.” (N2)*

### 3.5. Theme 3: Care Trade‐Offs Under Resource Constraints

This theme presents how nurses arranged care when time and staffing were insufficient to meet multiple concurrent demands. Participants described prioritizing urgent care and key treatments and continually revising their original plans as new tasks arose. These accounts concerned decisions about what could be completed first and what needed to be delayed within the resources available during a shift.

#### 3.5.1. Subtheme 3.1: Prioritizing Safety and Key Treatments

Under high‐workload conditions, nurses prioritized tasks related to vital signs, changes in patients’ conditions, and key treatments. They explained this ordering in terms of urgency and clinical risk. Care requiring more time for communication and explanation was sometimes delayed while these immediate needs were addressed.

*“…we deal with what’s urgent and important first, especially anything related to vital signs, changes in condition, and key medications… when it’s busy, we can only make sure these basics are covered first.” (N22)*

#### 3.5.2. Subtheme 3.2: Unexpected Tasks and Task Reordering

Nurses described how new clinical tasks or administrative duties interrupted their original work plans. Limited night‐shift staffing, unfinished tasks at handover, sudden changes in patients’ conditions, and last‐minute requests for materials or reports required repeated adjustments to the work sequence. Participants reported continually switching between tasks and sometimes working overtime to complete interrupted work.

*“On night shifts, we’re already short-staffed… a lot of things have to be handled at the same time… and in that situation, it’s just impossible to do everything thoroughly.” (N8)*

### 3.6. Theme 4: Emotional and Professional Experiences

Participants described emotional and professional experiences associated with inadequate care, uneven burdens, and sustained work pressure. These included guilt and lingering fear about potentially missed care, emotional strain in interactions, and a gradual shift toward completing essential duties and avoiding errors. These accounts reflected nurses’ experiences of their own working states and interpersonal relationships.

#### 3.6.1. Subtheme 4.1: Guilt and Lingering Fear

Nurses described repeatedly revisiting care episodes after busy shifts, particularly when they felt that checks or other care activities had not been sufficiently completed. Some participants worried that work interruptions might have caused them to overlook a task or risk. These accounts conveyed both guilt about care falling short of their expectations and lingering fear about potential consequences.

*“Before giving the medication, I didn’t really have time to fully check its effects… that day the workload was too heavy and time was tight… afterward I felt a bit guilty, because I knew the care wasn’t really sufficient.” (N2)*

#### 3.6.2. Subtheme 4.2: Emotional Buildup and Relationship Strain

Participants linked sustained workload pressure and repeated experiences of unfairness to irritability and frustration in communication. They described hurried exchanges, arguments, and difficulty processing their emotions, with tensions sometimes extending to interactions with colleagues, patients, and family members. These accounts focused on how accumulated emotions were expressed in interpersonal relationships.

*“When you’re rushed off your feet and communication isn’t going smoothly, it’s really frustrating… that sense of helplessness, powerlessness, even anger just comes out.” (N7)*

#### 3.6.3. Subtheme 4.3: Defensive Work Orientation

Under prolonged high pressure and persistent imbalance at work, some nurses gradually adjusted their work goals and psychological expectations. They reduced their pursuit of “doing better” and placed greater emphasis on completing basic tasks and avoiding errors. This change reflected an adjustment in their work engagement under long‐term pressure and represented a self‐protective work strategy.

*“Over time, people stop aiming for higher quality… and just focus on getting the basics done first.” (N8)*

### 3.7. Theme 5: Expectations for Task Allocation Reform

This theme presents nurses’ specific expectations for task allocation reform based on their everyday experiences under high‐workload conditions. The interviews showed that nurses hoped reform would align more closely with frontline practice and address core issues such as workload distribution, support arrangements, and the transparency of rules. They focused on whether task allocation could reflect patient complexity, nurses’ conditions, and work content, whether timely support could be provided during peak periods, and whether additional tasks could be distributed more evenly. For participants, the effectiveness of reform depended on whether work arrangements reflected actual work and were easier to understand and accept.

#### 3.7.1. Subtheme 5.1: Allocation Based on Actual Workload

Nurses hoped that task allocation would consider patient numbers, illness complexity, procedural risks, nurses’ competence, and physical capacity, so that the assigned work would reflect the demands they actually faced. A head nurse further suggested that workload assessment should give greater weight to illness severity and the proportion of high‐risk patients, while also considering holidays and consecutive working days.

*“…you need to factor in patient numbers, illness complexity… as well as nurses’ experience, professional competence, physical condition, and current workload… only then can task allocation better reflect the real workload and be fair.” (N3)*

#### 3.7.2. Subtheme 5.2: Flexible Support During Peak Periods

Nurses hoped to receive timely support during periods of concentrated demand, including morning treatments and shift handovers. They linked this support to more balanced workloads within a shift. A head nurse suggested retaining some flexibility alongside routine work to accommodate unexpected situations. These accounts emphasized access to additional help and room for adjustment when demand increased.

*“…during peak periods, there should be more staff added… so the workload can be more balanced… and the arrangements more fair.” (N2)*

#### 3.7.3. Subtheme 5.3: Clear Allocation and Rotation Rules

Nurses wanted stable, predictable allocation rules and regular rotation to reduce the repeated concentration of heavier burdens on the same individuals. A head nurse also emphasized the need for clear arrangements and a more balanced distribution of additional responsibilities, including research, inspections, and materials‐related work. These accounts focused on the transparency and consistency of allocation procedures.

*“…more importantly, it’s about task allocation—extra tasks like research, inspections, and materials… should be distributed more evenly… at least there should be clear arrangements.” (N12, head nurse)*

### 3.8. Theme 6: Concerns About Task Allocation Reform

This theme presents nurses’ concerns about the outcomes and practical conditions of task allocation reform alongside their hopes for change. The interviews showed that nurses held complex attitudes toward reform. They hoped that the current situation would improve but also worried that work demands and expectations would increase, adding to their actual burdens. They were concerned that insufficient resources would make reform difficult to implement and that frontline clinical nurses would ultimately bear the consequences. Experiences of prolonged high‐pressure work led many nurses to remain cautious about reform, hoping to see practical changes before deciding whether to offer greater trust and cooperation.

#### 3.8.1. Subtheme 6.1: Further Increases in Work Demands

Nurses worried that reform would lead to tighter task arrangements, more detailed requirements, and greater difficulty in securing rest. They were concerned that so‐called optimization, once implemented in frontline practice, could translate into a more intense work pace, less room to breathe, and additional pressure. This concern arose from their ongoing assessments based on previous work experiences and reflected frontline clinical nurses’ sensitivity to the practical consequences of reform.

*“…what I worry about most is that the reform might end up… with tighter schedules, less guaranteed rest, and even more pressure than before.” (N10)*

#### 3.8.2. Subtheme 6.2: Insufficient Resources for Implementation

Nurses worried that reform plans would be difficult to implement if staffing, time, and organizational support remained insufficient. A frontline clinical nurse expressed concern that the work would still fall on existing staff. A head nurse added that labor‐cost controls and insufficient resources could make proposed staffing increases difficult to achieve. Both accounts linked reform implementation to the resources needed to put the changes into practice.

*“Staff shortages and lack of resources are the real issue… no matter how good the reform plan sounds, without enough people to carry it out… it still falls on us.” (N18)*

#### 3.8.3. Subtheme 6.3: Caution About Reform Outcomes

Participants remained cautious about whether reform would be effective and hoped to see practical improvements before placing greater trust in it. A frontline clinical nurse focused on whether the changes would actually reduce the workload. A head nurse similarly wanted to see whether reform would protect time off and avoid adding to staff burdens. These accounts reflected participants’ wish to judge reform through actual changes in everyday work and rest.

*“…right now I’m a bit more cautious… whether it will really reduce the burden… if it’s just a change in wording, I wouldn’t feel very reassured.” (N25)*

## 4. Discussion

Taking task allocation as the entry point, this study explored nurses’ fairness experiences under high‐workload conditions in Chinese hospitals and identified six themes: fairness judgments in task allocation; fairness experiences and care adequacy; care trade‐offs under resource constraints; emotional and professional experiences; expectations for task allocation reform; and concerns about task allocation reform. Previous workload research has examined staffing constraints, nurses’ well‐being, and patient safety [4, 18, 19]. From an organizational justice perspective, this study further illustrates the multiple considerations underlying nurses’ evaluations of work arrangements, including whether support matched their workloads, whether their efforts received recognition and rewards, and whether allocation and rotation rules were reasonable. Alongside these judgments, they described concerns about care adequacy, emotional and professional experiences, and expectations and concerns about reform. Frontline clinical nurses provided the core accounts, while head nurses contributed supplementary information about ward‐level arrangements.

Fairness judgments in task allocation reflected nurses’ overall evaluations of workloads and the arrangements accompanying them. In accounts of demanding tasks concentrated among experienced nurses, participants understood the clinical reasons for assigning complex care to experienced staff but questioned the continued accumulation of burdens and limited rotation opportunities [20]. Their concerns centered on whether demanding tasks were shared reasonably and whether support was available to those undertaking them. Limited staff availability indicated that rostered staffing numbers did not fully reflect the staff capacity available for direct care, as teaching and other duties occupied nurses’ time. This is consistent with Benjamin et al.’s qualitative study, which found that nurses’ perceptions of workload were shaped not only by task demands but also by the availability of team support and opportunities to take breaks [21]. Meanwhile, workload overlooked by existing indicators reflected insufficient recording and recognition of activities such as communication and coordination, echoing previous research on the limitations of workload assessment [22]. Within fairness experiences and care adequacy, alignment between effort and rewards further concerned whether the responsibilities undertaken were matched by corresponding compensation and recognition. Care reduced under time constraints illustrated difficulties in delivering care within these work arrangements: during busy shifts, time for health education, communication, and psychological support was reduced, leaving some patient needs insufficiently addressed [23, 24]. A Swedish emergency nursing study reported similar shortcomings, with physical needs receiving more attention than relational and psychosocial needs [25]. Thus, fairness experiences and care adequacy captured nurses’ dual concerns during busy shifts: whether their efforts received corresponding rewards and recognition, and whether patients received the care they needed.

Care trade‐offs under resource constraints concerned how nurses managed multiple concurrent demands. Prioritizing safety and key treatments described decisions based on urgency and clinical risk, while unexpected tasks and task reordering reflected nurses’ repeated adjustments to existing work plans in response to new clinical demands or administrative requests [26]. This is consistent with Abdelhadi et al.’s qualitative study, which described how nurses weighed task priorities during work interruptions and decided which care activities to complete or defer according to their perceived controllability of the situation [27]. Emotional and professional experiences captured nurses’ feelings and changes in their work orientation within these situations. Guilt and lingering fear involved self‐blame when care fell short of nurses’ own expectations and subsequent worries about potentially overlooked tasks or risks. Previous research on moral distress also described nurses’ distress when resource constraints prevented them from providing care they considered necessary [28, 29]. Emotional buildup and relationship strain involved irritability, frustration, and tension in interactions with colleagues, patients, and family members [30, 31]. Defensive work orientation reflected adjustments to nurses’ work goals, with some shifting their focus toward completing basic tasks and avoiding errors. Skinner et al.’s qualitative study also described nurses reducing their effort and commitment following unfair treatment [32], resembling the changes in work investment reported in the present study. Emotional fatigue accompanying persistent clinical workload and organizational demands has also been documented in previous interviews with nurses [33]. These findings suggest that understanding nurses’ fairness experiences requires attention to task allocation and the conditions for fulfilling care responsibilities, alongside nurses’ emotional experiences and changes in their investment in work.

Nurses’ expectations for task allocation reform centered on workload assessment, timely assistance, and clearer rules. They called for allocation based on actual workload, taking into account patient numbers, illness complexity, procedural risks, and nurses’ competence and physical capacity. They also expected flexible support during peak periods to help meet temporary increases in care demands and sought clear allocation and rotation rules so that additional duties would be shared according to established procedures, with regular rotation. This is consistent with Holton et al.’s Australian rostering study, with both studies highlighting nurses’ emphasis on fairness and flexibility in work arrangements [34]. Head nurses added management considerations concerning workload assessment, room for adjustment within shifts, and the sharing of additional duties. Nurses’ concerns about task allocation reform centered on the possibility of additional burdens and constraints on implementation. They feared further increases in work demands, including tighter work schedules, more detailed requirements, and greater difficulty securing rest. They also worried about insufficient resources for implementation, specifically whether the staffing, time, and organizational support needed to implement changes would be available. Previous research on organizational transformation and nurses’ change fatigue has also addressed additional burdens and insufficient support during change [35, 36]. Participants also expressed caution about reform outcomes, wishing to see tangible improvements before placing greater trust in reform. These expectations and concerns suggest that changes in allocation rules require corresponding resources, with less visible work such as communication and coordination recognized and shared, and peak‐period assistance, rotation, compensation, and protected rest incorporated into specific shift arrangements [37]. Future research could follow the implementation of task allocation reform over time, examining changes in workload, the sharing of responsibilities, and opportunities for rest alongside nurses’ fairness experiences. Particular attention should be paid to how allocation rules are applied across shifts and whether the necessary staffing and support are available, to identify the conditions under which reform improves fairness experiences and the barriers to its implementation.

## 5. Limitations

This study has several limitations. First, convenience sampling recruited 27 female nurses from seven tertiary Grade A hospitals in four regions of China. Male nurses and nurses working in secondary hospitals or primary healthcare institutions were not represented. Potential selection bias and the restricted sample coverage limit the transferability of the findings. Second, the sample included 24 frontline clinical nurses and three head nurses. Interviews with head nurses supplemented the accounts of ward‐level work arrangements, but their small number limited the diversity of managerial perspectives represented. Third, data were collected through online interviews without direct observation of task allocation or care delivery. Participants’ accounts may have been influenced by recall bias and concerns about discussing workplace experiences. Judgments about care adequacy also relied solely on nurses’ perceptions, without supplementary patient accounts or direct observations. Fourth, the interview guide focused on predefined areas of inquiry, which may have shaped the scope and organization of the thematic analysis. Although open coding was used, experiences and issues beyond these areas may not have been fully explored. Finally, expectations and concerns about task allocation reform reflected participants’ views of potential changes. The study did not follow reform implementation and therefore could not determine whether these changes would improve nurses’ fairness experiences. Future research could track workload, support, and opportunities for rest following reform implementation, alongside nurses’ experiences, to examine whether reform addresses their concerns in practice.

## 6. Conclusion

This study described nurses’ fairness experiences under high‐workload conditions using task allocation as the entry point. Participants evaluated work arrangements in relation to the distribution of demanding tasks, actual staff availability, recognition of workload, and alignment between effort and rewards. They also described concerns about care adequacy, care trade‐offs under resource constraints, and emotional and professional experiences. Their expectations and concerns about task allocation reform highlighted allocation based on actual workload, flexible support during peak periods, and clear allocation and rotation rules, alongside attention to implementation resources and the protection of rest. These findings provide a basis for considering task allocation arrangements that respond to nurses’ expressed needs.

## Author Contributions

**Yucheng Cao**: conceptualization (equal); data curation (supporting); formal analysis (equal); validation (lead); methodology (equal); writing–original draft preparation (supporting); project administration (lead); supervision (lead); writing–review and editing (supporting); investigation (equal); visualization (supporting).

**Lv Wu**: formal analysis (equal); methodology (equal); software (supporting); validation (supporting); writing–review and editing (lead).

**Yang Tang**: conceptualization (equal); data curation (lead); formal analysis (equal); methodology (equal); software (lead); writing–original draft preparation (lead); investigation (equal); visualization (lead).

**Xu Deng**: conceptualization (equal); data curation (supporting); formal analysis (equal); methodology (equal); writing–review and editing (supporting); investigation (equal).

**Tingting Yan**: project administration (supporting).

**Shaolong Zhou**: conceptualization (supporting); formal analysis (supporting); methodology (supporting); project administration (supporting); writing–review and editing (supporting).

**Yu Gao**: conceptualization (equal); data curation (supporting); formal analysis (equal); validation (supporting); methodology (equal); software (supporting); writing–original draft preparation (supporting); project administration (supporting); supervision (supporting); investigation (equal); visualization (supporting); writing–review and editing (supporting).

**Lili Deng**: conceptualization (equal); formal analysis (equal); validation (supporting); methodology (equal); project administration (supporting); supervision (supporting); writing–review and editing (supporting).

## Funding

This research received no specific grant from any funding agency in the public, commercial, or not‐for‐profit sectors.

## Ethics Statement

This interview study was approved by the Medical Ethics Committee of Yichun University (approval number: 2026074; effective date: February 1, 2026). Before each interview, the researcher explained to participants the study purpose, study content, interview format, potential risks, expected benefits, recording arrangements, scope of data use, and their rights, and obtained electronic informed consent. Participants were free to refuse to answer any question or withdraw from the study at any time before or during the interview, without providing any reason.

## Conflicts of Interest

The authors declare no conflicts of interest.

## Supporting Information

Additional supporting information can be found online in the Supporting Information section.

## Supporting information


**Supporting Information** Supporting S1. Semistructured Interview Guide. This supporting file provides the semistructured interview guide used for data collection, including questions concerning participants’ work situations, perceptions of fairness, task allocation under high‐workload conditions, the impacts of fairness or unfairness experiences, and expectations and concerns regarding task allocation reform.

## Data Availability

The data that support the findings of this study are available from the corresponding author upon reasonable request.
