# Supplementary material for: Task Allocation Under High Workload: A Qualitative Study of Nurses’ Fairness Experiences in Chinese Tertiary Grade A Hospitals
Source: J Nurs Manag. 2026 Oct 18;2026:2315457. doi: 10.1155/jonm/2315457 (PMC13678287; doi:10.1155/jonm/2315457)
Supplement: Supplementary file 1 [file JONM-2026-2315457-s001.docx]

**Supplementary S1. Semi-Structured Interview Guide**

Q1. Please briefly introduce your current work situation, including the hospital level and department, years of work experience, professional title, and your main work responsibilities at present.

Q2. In your daily work, how do you understand “fairness”? In your view, in what aspects is fairness in nursing work usually reflected?

Q3. In a specific shift, how are patients and tasks usually allocated to each nurse? Do you think this allocation method is reasonable in terms of workload, risk, and responsibility?

Q4. In your view, which tasks or responsibilities are more likely to be concentrated among a small number of nurses? How does this situation make you feel?

Q5. When the workload is particularly heavy and tasks are numerous, what do you usually prioritize first? Which aspects are often compressed, delayed, or cannot be completed to an ideal standard?

Q6. When a shift is particularly busy, do you feel that some things are difficult to balance at the same time? Could you describe this based on your actual experience?

Q7. Under what circumstances are you most likely to clearly feel “unfairness”? Could you give one or two memorable examples?

Q8. How do these experiences of fairness or unfairness affect you personally?

Q9. In your view, how do these situations affect patients and the team?

Q10. If task allocation were to be improved, what kinds of approaches would make you feel more reasonable and acceptable?

Q11. What do you most expect from task allocation reform?

Q12. What are your main concerns regarding task allocation reform?

Q13. If you could tell management in a few sentences, what do you think most needs to be changed at present?

Q14. Regarding fairness, task allocation, and care quality in nursing work, are there any important points that you did not have time to mention but consider very important?

**Summary of interview content — Corresponding questions**

Basic work situation of participants — Q1

Understanding of fairness in nursing work — Q2

Allocation of patients and tasks within specific shifts and perceived reasonableness — Q3, Q4, Q7

Manifestations of task concentration, responsibility distribution, and compression of care content under high-workload conditions — Q5, Q6

Impact of fairness or unfairness experiences on nurses, patients, and teams — Q8, Q9

Expectations and concerns regarding task allocation improvement — Q10, Q11, Q12, Q13

Other supplementary experiences and perspectives — Q14
